# The effects of the climatic elements on occurrence of the spontaneous pneumothorax

**DOI:** 10.1186/1749-8090-8-S1-P153

**Published:** 2013-09-11

**Authors:** SJ Haam, SS Lee, DY Lee, HC Paik

**Affiliations:** 1 Department of Thoracic and Cardiovascular Surgery, Gangnam Severance Hospital, Yonsei University, College of Medicine, Seoul, Korea

## Background

The rupture of bullas causes the spontaneous pneumothorax (SPM). But, it is uncertain which factors are associated with the rupture of bullas. We assessed the relationship between occurrence of SPM and climatic elements.

## Methods

We evaluated SPM patients who visited our institute and were between 15 and 45 years old from 2009 and 2011. We excluded the patients who were transferred from distant local area and had the vague occurrence day. Climatic elements contained temperature, humidity, wind velocity, atmospheric pressure and weather that the national meteorological administration provided.

## Results

342 of the patients were male and 42 were female. Mean age was 21.9±0.5 years old. Month (p=0.563), season (p=0.264) and weather (p=0.327) were not associated with the occurrence rate of SPM. Mean temperatures, mean humidity, mean wind velocities of SPM occurrence day and non-occurrence day were 12.4±10.5°C : 12.8±10.5°C (p=0.504), 61.8±15.5% : 60.9±15.0% (p=0.372), 2.6±1.0m/s : 2.6±2.1m/s (p=0.893) respectively. As to the atmospheric pressure, mean, maximum, minimal value of occurrence day and non-occurrence day were 1012.6±9.7 hPa : 1012.7±9.0 hPa (p=0.850), 1018.7±8.2 hPa : 1018.5±7.8 hPa (p=0.645), 1013.4±8.1 hPa : 1013.6±7.9 hPa (p=0.755) respectively. But, differences between maximum and minimum value were 5.3±2.9 hPa on occurrence day and 4.9±2.6 hPa on non-occurrence day (p=0.037).

## Conclusion

There was no relationship between the occurrence of SPM and weather, season, temperature, humidity and wind velocity. But, the difference of the atmospheric pressure throughout the day was associated with occurrence of SPM. We concluded that the change of atmospheric pressure influenced the rupture of bullas that caused SPM.

